# Romantic love evolved by co-opting mother-infant bonding

**DOI:** 10.3389/fpsyg.2023.1176067

**Published:** 2023-10-17

**Authors:** Adam Bode

**Affiliations:** School of Archaeology and Anthropology, ANU College of Arts and Social Sciences, The Australian National University, Canberra, ACT, Australia

**Keywords:** attachment, attraction, co-option, evolution, mother-infant bonding, pair bonding, romantic love

## Abstract

For 25 years, the predominant evolutionary theory of romantic love has been Fisher’s theory of independent emotion systems. That theory suggests that sex drive, romantic attraction (romantic love), and attachment are associated with distinct neurobiological and endocrinological systems which evolved independently of each other. Psychological and neurobiological evidence, however, suggest that a competing theory requires attention. A theory of co-opting mother-infant bonding sometime in the recent evolutionary history of humans may partially account for the evolution of romantic love. I present a case for this theory and a new approach to the science of romantic love drawing on human psychological, neurobiological, and (neuro)endocrinological studies as well as animal studies. The hope is that this theoretical review, along with other publications, will generate debate in the literature about the merits of the theory of co-opting mother-infant bonding and a new evolutionary approach to the science of romantic love.

## 1. Introduction

For almost 25 years, Fisher’s (1998, 2000) and Fisher et al.’s (2002) theory of independent emotion systems has provided the predominant evolutionary account of romantic love; however, a theory of co-opting mother-infant bonding (see Bode and Kushnick, 2021) may better help to explain aspects of romantic love’s evolutionary history and mechanisms. This review presents the case for a theory of co-opting mother-infant bonding in the evolutionary history of romantic love by merging proximate (i.e., mechanistic) and ultimate (i.e., evolutionary) perspectives (see Hofmann et al., 2014; Zietsch et al., 2020) and proposes a new approach to the science of romantic love based on this theory.

First, I introduce two theories that have informed the theory of co-opting mother-infant bonding in the evolutionary history of romantic love: Fisher’s (1998) theory of independent emotions systems and the brain opioid theory of social attachment (BOTSA; see Machin and Dunbar, 2011). Second, I define relevant terminology, present a brief history of the theory of co-opting mother-infant bonding, outline the evidence for this theory with reference to psychological, neurobiological, and (neuro)endocrinological studies, and introduce the preliminary evidence supporting the theory. Third, I outline the basic premise of the theory with specific consideration of the evidence supporting the idea that romantic love involves activity of both the bonding attraction and attachment systems. Fourth, I introduce a new model of romantic love that details the evolutionary history, mechanisms, and psychological outputs of romantic love. Fifth, two unanswered questions about the evolutionary history of romantic love are posed. I conclude with ideas for future research. The result is a preliminary justification for the theory of co-opting mother-infant bonding in the evolutionary history of romantic love, justification of a new approach to the science of romantic love, and a *de facto* critique of Fisher’s (1998) outdated model.

### 1.1. Purpose of this article

The scientific literature around romantic love is limited. There have only been about 45 empirical studies of the biology of romantic love (Bode and Kowal, 2022). The number of studies specifically investigating the psychology of romantic love is probably in the same vicinity (although a large number do include romantic love as a variable of interest). The research is piece-meal or simple replications of previous studies (i.e., specifically in relation to fMRI studies). No overarching theory guides research into romantic love and this leaves the science of romantic love lacking. Romantic love is under-researched given its importance in family and romantic relationship formation, its influence on culture, and its proposed universality (see Jankowiak and Fischer, 1992; Buss, 2019a, 2019b; Bode and Kushnick, 2021).

While I recognize that the topic of this article will be of interest to a broad audience, my target audience is individuals involved in romantic love research. This includes, primarily, biologists and psychologists. My purpose is three-fold: (i) outline the preliminary evidence for the theory of co-opting mother-infant bonding, (ii) provide guidance for future research based on a coherent theory that can be tested, and (iii) serve as a critique of Fisher’s (1998) theory of independent emotions systems. In my opinion, Fisher’s (1998) theory not only mischaracterizes the mechanisms of romantic love, but fails to accurately articulate the processes through which romantic love emerged, evolved, and functions.

I outline the history of the theory of co-opting mother-infant bonding in the evolutionary history of romantic love below. It has most recently been articulated by Bode and Kushnick (2021), but that effort was limited in scope and evidence, drawing on only a few relevant human studies. This current article extends on that attempt by drawing on the full breadth of human research of romantic love and insights from extensive animal research on pair bonding. I provide ideas for future research at the end of this article. Those propositions are informed by a theory that helps to explain not only the evolutionary history of romantic love, but also its functions and mechanisms. I also provide a strident critique of Fisher’s (1998) theory with the aim of correcting the peer reviewed scientific record (I note Fisher has published popular science books in addition to a relatively small number of peer reviewed articles on the topic, but these cannot be considered academic texts). Fisher’s (1998) theory is well known, but has failed to generate hypotheses or guide research. The useful concepts and ideas it provides should be adopted while the general theory should be discounted as an accurate representation of the evolution or mechanisms of romantic love.

## 2. Two theories that inform the theory of co-opting mother-infant bonding in the evolutionary history of romantic love

### 2.1. Fisher’s theory of independent emotion systems

Fisher’s (1998, 2000) and Fisher et al.’s (2002) theory of independent emotions systems describe sex drive (lust), attraction (romantic love), and attachment (pair bonding). Fisher contends that these three systems evolved separately to serve specific functions. Sex drive is associated with estrogens and androgens and motivates individuals to engage in sexual activity. Attraction is associated with catecholamines (i.e., dopamine, norepinephrine), phenylethylamines, and serotonin and focuses efforts on preferred mating partners. Attachment is associated with oxytocin and vasopressin and enables individuals to engage in parental care. She concludes that “…during the course of hominid evolution, these three emotion circuits-lust, attraction, and attachment-became increasingly independent from one another […]” (Fisher, 2000, p. 97). The great strength of Fisher’s theory is that it describes three distinct systems involved in mammalian reproduction.

Despite being rational, well informed for the time, clearly articulated, and appealing because of its simplicity, Fisher’s (1998, 2000) and Fisher et al.’s (2002) theory was created and developed without the ample psychological, neurobiological, and endocrinological evidence available to today’s researchers. In the absence of that type of evidence, Fisher draws on theorizing in evolutionary biology, psychological research, and a general understanding of hormones and neurotransmitters to generate the theory (Fisher, 1998). Later iterations incorporated findings from one neuro-imaging study to support the hypothesis that “…romantic love is associated with a discrete constellation of neural correlates and distinct from the neural systems associated with the other primary mating emotion–motivation systems: lust and attachment […] (Fisher et al., 2002, p. 417).” In a follow-up article, Fisher (2006) draw on a small number of neuroimaging studies to reinforce the point that romantic love, like the behavioral attraction system in mammals, is associated with the dopaminergic reward pathway. A subsequent publication by Fisher et al. (2016) further articulates aspects of the theory. Fisher’s (1998, 2000) and Fisher et al.’s (2002) theory does not provide any detailed explanation as to how these three systems evolved (in peer reviewed articles) except to say that they evolved separately and exist in mammals (and attraction is common to all mammals; Fisher, 1998).

While I disagree with the basic premise of Fisher’s (1998) model, I think there is some utility in recognizing the role of distinct but interrelated systems. I believe the systems involved in romantic love differ from those posed by Fisher, and the initial notion that these three systems became increasingly separated has actually been contradicted by her later work (Fisher et al., 2016). I do, however, see great value in using the basic idea that romantic love involved distinct pre-existing systems that interact (see Bode and Kushnick, 2021).

### 2.2. Brain-opioid theory of social attachment

The *brain opioid theory of social attachment* [see Machin and Dunbar, 2011 for review of evidence; see also Panksepp, 1981 (cited in Panksepp, 2005) for genesis] contends that endogenous opioids play an important role in the full spectrum of social attachment. It has its origins in studies demonstrating behavioral and emotional similarities between individuals involved in intense, close relationships and those using narcotics (e.g., Liebowitz, 1983; Panksepp, 1999 cited in Machin and Dunbar, 2011).

Earlier accounts of the similarities between intense, close social relationships and narcotics addiction included euphoria, tolerance-habituation, and withdrawal (see Liebowitz, 1983 cited in Machin and Dunbar, 2011). More recently developed understandings of addiction suggest another manner of viewing the similarities might be through the three stages of binge/ intoxication, withdrawal/ negative affect, and preoccupation/ anticipation (Koob and Volkow, 2016; see also Bode and Kushnick, 2021). Both approaches help to explain the processes involved in intense, close relationships.

One endogenous opioid postulated to play a particularly important role in intense, close relationships is *β*-endorphin (Machin, 2022). It is believed to play an important role in the pain of social isolation and reward of social contact (Panksepp et al., 1997; see also Machin and Dunbar, 2011 for succinct summary). There is preliminary evidence of *β*-endorphins involvement in new parents and the early stages of a romantic relationship (Ulmer-Yaniv et al., 2016) as well as other social relationships in non-primate mammals, non-human primates, and humans (Machin and Dunbar, 2011).

*β*-endorphin- releasing neurons are found in high densities in a number of regions associated with mother-infant bonding and romantic love, such as the hypothalamus and mesolimbic structures involved in reward (Bodnar and Klein, 2006). Receptors are also found in regions associated with both states, including the basal ganglia and corticolimbic regions (Stefano et al., 2000). Endogenous opioids interact with a number of other neurotransmitters and hormones implicated in romantic love, such as dopamine, serotonin, and testosterone (see Machin and Dunbar, 2011).

The BOTSA provides preliminary evidence for the involvement of the endogenous opioid system and related systems in multiple forms of love, including mother-infant bonding and romantic love. It also highlights the mechanistic similarities between all forms of close social relationships, including forms of love, all of which involve the attachment system.

## 3. A theory of co-opting mother-infant bonding

### 3.1. Terminology

#### 3.1.1. Mother-infant bonding/maternal–infant bonding

The concept of mother-infant bonding is often superficially developed and subject to confusion with related concepts (Bicking Kinsey and Hupcey, 2013). It is synonymous with “maternal–infant bonding” and is characteristic of mammals (see Broad et al., 2006; Numan and Young, 2016). One definition of maternal–infant bonding, as it relates to humans, is:

[…] a maternal-driven process that occurs primarily throughout the first year of a baby’s life, but may continue throughout a child’s life. It is an affective state of the mother; maternal feelings and emotions toward the infant are the primary indicator of maternal–infant bonding. Behavioral and biological indicators may promote maternal–infant bonding or be an outcome of maternal–infant bonding, but are not sufficient to determine the quality of maternal–infant bonding nor are these indicators unique to the concept (Bicking Kinsey and Hupcey, 2013, pp. 9–10).

Bowlby (1969/1982) is more precise in his assessment of when mother-infant bonding is occurring. He suggests that infants start to exhibit attachment behaviors by 6 months of age and are generally exhibiting the full breadth of attachment behaviors by 9 months of age. Following this development, interaction between the mother and infant is bi-directional, and therefore it can be inferred that mother-infant bonding is particularly important in the first 9 months of a child’s life.

Mother-infant bonding appears to involve three distinct systems: a reward and motivation (*bonding attraction*) system, an oxytocin and vasopressin related (*attachment*) system, and an *obsessive thinking* system. The bonding attraction systems is associated with oxytocin and dopamine systems (Rigo et al., 2019; Shih et al., 2022), and is characterized by activity in the left ventral tegmental area (VTA), right thalamus, left substantia nigra, and putamen (Shih et al., 2022) among other regions (see Rigo et al., 2019). The attachment system is associated with dopamine, oxytocin, and opioids (see Numan and Young, 2016 for discussion of mammals), and is characterized by circulating oxytocin (Ulmer-Yaniv et al., 2016; Scatliffe et al., 2019) and opioids (Ulmer-Yaniv et al., 2016), among other factors, in humans. Nothing is known about the mechanisms contributing to obsessive thinking, but it is associated with preoccupation with the infant (Kim et al., 2013).

#### 3.1.2. Romantic love

A comprehensive, ethologically-informed definition of romantic love is:

[…] a motivational state typically associated with a desire for long-term mating with a particular individual. It occurs across the lifespan and is associated with distinctive cognitive, emotional, behavioral, social, genetic, neural, and endocrine activity in both sexes. Throughout much of the life course, it serves mate choice, courtship, sex, and pair-bonding functions. It is a suite of adaptations and by-products that arose sometime during the recent evolutionary history of humans (Bode and Kushnick, 2021, p. 21).

Unless indicated otherwise, in this review, “romantic love” refers to the state that usually occurs in the early stages of a romantic relationship (i.e., early-stage romantic love) and not long-term romantic love (see Acevedo and Aron, 2009; O’Leary et al., 2011; Acevedo et al., 2012 for explanation of long-term romantic love). Romantic love is sometimes referred to as “passionate love,” in the field of psychology (Feybesse and Hatfield, 2019). I use the term “romantic love” because that tends to be the name conferred in the fields of biology (see Bode and Kushnick, 2021; Bode and Kowal, 2022). Romantic love precedes companionate love (see Hatfield and Walster, 1985), which is the love felt less intensely among individuals in an established pair bond. It tends to last up to about 2-to-3 years and is characteristic of the early stages of a romantic relationship (see Tennov, 1979; Marazziti et al., 1999; Marazziti and Canale, 2004; Emanuele et al., 2006). It is evolutionarily, mechanistically, and psychologically distinct from attachment. Throughout this article, all studies related to “romantic love” have been undertaken in humans.

#### 3.1.3. Attraction system(s)

Fisher (1998) argues that the attraction system serves to focus energies on a preferred mating partner. I, however, believe that what Fisher is referring to can better be described in terms of two separate systems with distinct functions. I would suggest that one system could be best termed *courtship attraction*, which is a system that may be characterized by activity of dopamine, oxytocin, and opioids (see Fisher, 1998; Baskerville and Douglas, 2010; Meier et al., 2021), and which focuses energies on preferred mating partners for the purpose of facilitating copulation – essentially, mate choice. This most closely aligns with Fisher’s (1998) conceptualization of attraction and would be characteristic of the phenomenon of love at first sight, something which could better be termed an *acute courtship attraction episode* (see Zsok et al., 2017 for distinction with romantic love), or a crush. Unfortunately, the fMRI studies that measure neural activity in people experiencing romantic love would be measuring primarily, bonding attraction (introduced below), and only possibly courtship attraction. As a result, we know very little about the neural and endocrine characteristics of courtship attraction. Courtship attraction is common to all mammalian species (see Fisher, 1998; Fisher et al., 2006) as well as other classes of animals that sexually reproduce.

The second type of attraction, and something overlooked by Fisher (1998), could best be termed *bonding attraction*. It differs from courtship attraction in that it tends to occur during or following the establishment of a bond and serves to focus energies on a bonded object. Bonding attraction is characterized by a strong desire for proximity with the bonded object and separation distress when that proximity is interrupted (see Flower and Weary, 2001), which may be attributable to a distinct panic/ separation distress system (Panksepp et al., 1997; see also Panksepp, 2005 for summary) which may have been subsumed into the mammalian bonding attraction system. Bonding attraction, too, is probably characterized by dopamine, oxytocin, and opioid activity (arguments made in the sections below; see Baskerville and Douglas, 2010; Rigo et al., 2019; Shih et al., 2022; see also Panksepp et al., 1997; Preter and Klein, 2008; see also Panksepp, 2005) and would be common to all mammals, at least females, and to both sexes of species that pair bond, including humans. It would also be common to species in other classes that bond, such as most birds.

#### 3.1.4. Attachment system

The attachment system is the system responsible for forming and maintaining very close personal relationships (e.g., caregiver-child and romantic relationships). It is commonly associated with oxytocin and vasopressin (Carter, 2017a,b) although other neurotransmitter systems (e.g., opioids) must play a role. Early theorists considered it only in terms of caregiver-infant relationships and suggested it served the function of promoting proximity between caregiver and child, ensuring safety (see Bowlby, 1969/1982); later theorists associated it with romantic relationships and pair bonds (see Hazan and Shaver, 1987; Shaver et al., 1988; Fraley and Shaver, 2000; see also Fraley et al., 2005). The attachment system plays a role in other less intense, but nonetheless close, social relationships, sometimes referred to as bonds (see Carter, 2017b) as well as the very strong bond an infant displays towards its attachment figures (see Barbaro, 2020).

#### 3.1.5. Co-option

Co-option is a process whereby a trait (e.g., mechanism, morphology, behavior) in a species is replicated to serve a different function to that which it originally served (McLennan, 2008; see also Ganfornina and Sánchez, 1999; True and Carroll, 2002). A commonly cited example is the feathers of a bird that may have originally served a means of thermal regulation but later were produced in a morphologically innovated form (Prum, 2005) to serve a role in flight (see Bergstrom and Dugatkin, 2016). Similar processes can occur in psychological traits. Co-option is sometimes narrowly associated with “exaptation.” “The former [a process] refers to the fact that parts used in the formation of a new [trait] can be recruited from pre-existing features. The latter [in evolutionary biology] is understood to mean that functional change is possible with structural continuity” (Bozorgmehr, 2020, p. 2). To clarify, exaptation is when a trait retains its original form but takes on a new function; co-option is the process whereby any trait takes on a new function, regardless of whether the original form is retained or not (McLennan, 2008). An analogy would be that exaptation is akin to using an umbrella as a parasol whereas co-option occurs regardless of whether we are talking about an umbrella used as a parasol or a bucket pierced with holes to make a watering can. Phylogenetic analysis can sometimes be used to distinguish traditional adaptions from co-option (or exaptation) by determining if a particular function has preceded, accompanied, or followed evolution of a particular function (see Blackburn, 2002). An original psychological characteristic must have preceded the secondary psychological characteristic for co-option to have occurred.

#### 3.1.6. The theory of co-opting mother-infant bonding in the evolutionary history of romantic love

I believe that the theory of co-opting mother infant bonding in the evolutionary history of romantic love can be articulated as a theory that suggests that some of the cognitions, emotions, behaviors, neurobiology, (neuro)endocrinology, and genetics of mother-infant bonding were co-opted to form romantic love (see Bode and Kushnick, 2021). The function changed from mother-infant bonding to pair bonding (i.e., pair bond formation). Other mechanisms and psychology (i.e., courtship attraction and sexual desire) may have also been combined and modified (see Bode and Kushnick, 2021), but continued to serve their pre-existing functions, although expressed differently.

### 3.2. A brief history of the theory of co-opting mother-infant bonding

There are several important stages in the development of the theory that romantic love evolved from co-opting mother-infant bonding: animal research, early human research, comprehensive reviews, and articulation of the theory. Originally, many animal studies highlighted the similarities between mother-infant bonds and other social relationships, including pair bonds. These studies are summarized in multiple reviews (e.g., Carter, 1998; Carter and Keverne, 2002; Young and Wang, 2004; Broad et al., 2006; Numan and Young, 2016; Walum and Young, 2018) and tend to focus on mammals, notably rodents.

Leckman and Mayes (1999) conducted an analysis of the similarities between romantic love and early parental love (love of a parent for their offspring, and of which mother-infant bonding is one component). They found substantial similarities between the two types of love. Bartels and Zeki (2004), famously, conducted a study comparing the neurobiological characteristics of romantic love and maternal love. By maternal love, I mean the love a mother feels for her offspring. They showed that some brain regions implicated in romantic love overlap precisely with those involved in maternal love. They emphasized similarities in regions rich with oxytocin and vasopressin receptors. A subsequent meta-analysis of the literature about love (Ortigue et al., 2010) demonstrated substantial similarities between types of love (including romantic love and maternal love) in terms of activity in dopamine-rich structures. Recently, a meta-analysis of maternal and romantic love using an activation likelihood estimation (Shih et al., 2022) was undertaken. That meta-analysis highlighted similarities between maternal and romantic love in the dopamine-rich left VTA. It also acknowledged similar networks of affective and motivational processing were distributed differently in the two types of love. The authors specifically suggested that their findings support the notion of “common evolutionary roots” in both maternal and romantic love.

A number of notable articles have reviewed the animal evidence that the proximate mechanisms that cause mother-infant bonding play a role in other social relationships, including pair bonds (see Bales et al., 2021 for a discussion of the definitions of pair bond; see also Kushnick, 2022, June 4). Carter and Keverne (2002) identified maternal–infant bonding as an appropriate animal model for studying the biological basis of pair bonding. Young (2003) first noted, “[i]t is conceivable that similar neural and molecular mechanisms that have evolved for regulating the mother-infant bond have been co-opted to produce the pair bond” (p. 94). He emphasized the role of oxytocin in both of these bonds. Young and Wang (2004) also proposed the notion that there is similar neural and endocrinological oxytocin activity in mother-infant bonds and pair bonds. Broad et al. (2006) described shared neural and hormonal mechanisms across a range of social relationships in animals.

Numan and Young (2016) explicitly described the similarities between the mechanisms involved in mother-infant bonding and pair bonding in rodents. They suggested that “the neural circuitry and mechanisms that underpin the long-term mother–infant attraction may have provided a primordial neural scaffold” (Numan and Young, 2016, p. 99) upon which pair bonds evolved. Walum and Young (2018) outlined what is known from animal research about the mechanisms causing the formation and maintenance of pair bonds. They argue that the pair bond and romantic love may be coexistent; “[…] pair bonding is the evolutionary antecedent of romantic love and […] the pair bond is an essential element of romantic love” (Walum and Young, 2018, p. 12). Numan (2020) argued how the mother-infant bond potentially provides the neural foundation for the enduring pair bond that forms between mates in socially monogamous mammalian species.

Some notable publications have played an important role in advancing knowledge of the theory that romantic love evolved by co-opting mother-infant bonding. Diamond (2008; see also Diamond et al., 2010) articulates a process through which this evolution occurs. She states, with confidence, that adult pair bonding is an “exaptation” (Diamond, 2008, p. 226). While I would suggest that romantic love is a suite of adaptations and by-products (Buss, 2019a, 2019b; Bode and Kushnick, 2021), rather than an exaptation, this does support the claim that mother-infant bonding and romantic love are mechanistically and evolutionarily linked through a process of co-option. Later, Young and Alexander (2012) published a popular science book that described the process of co-option in terms a lay person could understand. These were followed in 2021 by a review article (Bode and Kushnick, 2021) on the biology of romantic love that drew on a small number of studies of romantic love, maternal love, and parental love to suggest that romantic love evolved by co-opting mother-infant bonding mechanisms. That review was the first to suggest the theory is as important in understanding the evolution of romantic love as Fisher’s (1998, 2000) and Fisher et al.’s (2002) theory of independent emotion systems, and even suggested the two theories could complement each other.

### 3.3. Evidence supporting a theory of co-opting mother-infant bonding in humans

This sub-section introduces evidence for the co-option of mother-infant bonding mechanisms in the evolutionary history of romantic love. I do this by providing context to Fisher’s (1998, 2000) and Fisher et al.’s (2002) theory of independent emotion systems and two lines of evidence directly supporting the theory of co-opting mother-infant bonding: psychological and neurobiological/ (neuro)endocrinological similarities between romantic love and mother-infant bonding. I focus on human studies rather than animal research because pair bonding evolved in different mammals independently of each other (see Fraley et al., 2005), rather than as part of a single evolutionary history (as evidenced by no ancient common ancestor to all mammals that pair bond). The phylogenetic distance between humans and the primary species used as models to investigate pair bonds (i.e., prairie voles) means that most of the comparative animal studies found in the literature are less informative than human studies. This is because we know that certain common structures in primates and rodents can contain different receptors (Zablocki-Thomas et al., 2022). While inferences can be made about romantic love in humans from animal models (see Fischer et al., 2019), far greater certainty about the mechanisms of romantic love is gained from considering human research.

Most of the human biological evidence (see Bode and Kowal, 2022 for a comprehensive list of neuroimaging, endocrinological, and genetics studies investigating romantic love) comes from cross-sectional studies using repeated measures designs of people experiencing romantic love or comparing groups experiencing romantic love with groups not experiencing romantic love (i.e., singles or people in a relationship for a long time). Seminal fMRI studies tend to ask participants in love to view an image of their loved one and compare this with viewing an image of a familiar other, although other designs and stimuli do exist (see Bode and Kowal, 2022). Endocrinological studies tend to measure circulating peptides in blood in a group of individuals experiencing romantic love and a control group of individuals not experiencing romantic love, although a small number of endocrinological studies (i.e., Marazziti et al., 1999; Marazziti and Canale, 2004; Emanuele et al., 2006) have identified these differences using longitudinal methods.

There is utility in drawing on animal research when considering pair bonding and I therefore do use animal evidence to supplement human evidence. Readers are referred to three reviews on pair bonding described above (i.e., Broad et al., 2006; Numan and Young, 2016; Walum and Young, 2018) for comprehensive accounts of animal research related to this topic and an interesting review by Zablocki-Thomas et al. (2022) which provides some neurobiological comparisons between love in humans and animals. I believe there is sufficient human evidence to make a case for the theory of co-opting mother-infant bonding in the evolutionary history of romantic love. It appears that romantic love displays some of the same psychological characteristics as mother-infant bonding, uses some of the same, or similar, mechanisms, but serves different functions (see McLennan, 2008).

#### 3.3.1. Mother-infant bonding preceded the evolution of romantic love

Before outlining the psychological and mechanistic evidence that romantic love evolved by co-opting mother-infant bonding, it is necessary to first determine whether mother-infant bonding preceded the evolution of romantic love (see Blackburn, 2002). In a review of the biology of romantic love, Bode and Kushnick (2021) consider the evolutionary history of romantic love with reference to pair bonding. They suggest that romantic love evolved in concert with pair bonds and describe the phylogenetic relationships among select mammal species that pair bond. In a phylogenetic cladogram, they place the established origin of mother-infant bonding physiology at the common ancestor of all mammals. This is supported by the fact that all mammals present varying degrees of maternal care, including, especially, the feeding of young with milk (see Pond, 1977), and responsiveness towards infants (Rilling and Young, 2014). These behaviors were present in the earliest mammals, dated to about 200 million years ago (Upham et al., 2019; Álvarez-Carretero et al., 2022). As such, mother-infant bonding preceded the evolution of romantic love (as pair bonding in the human lineage did not evolve until much later; see Fisher et al., 2016; Bode and Kushnick, 2021). It is therefore appropriate to consider the remaining evidence for the co-opting of mother-infant bonding in the evolution of romantic love.

#### 3.3.2. Romantic love is a suite of adaptations and by-products

Fisher’s (1998, 2000) and Fisher et al.’s (2002) theory of independent emotion systems states that the attraction system is, essentially, responsible for romantic love, and that it is associated with the catecholamines (i.e., norepinephrine and dopamine), phenylethylamine, and serotonin. Indeed, research has demonstrated changes in serotonergic and dopaminergic systems in people experiencing romantic love (Marazziti et al., 1999; Langeslag S. J. E. et al., 2012; Marazziti et al., 2017). Fisher takes a narrow definition of romantic love (the attraction system) that suggests romantic love serves to focus energies on a preferred mate. Others, however, frame romantic love in terms of being a suite of adaptations and by-products (Bode and Kushnick, 2021; see also Buss, 2019a, 2019b). This suggests that romantic love serves additional broad (i.e., sex and pair bond formation) and specific (i.e., providing sexual access, signaling sexual fidelity, providing psychological and emotional resources, displaying commitment, and providing signals of parental investment) functions.

The mechanisms of romantic love are more complicated than that proposed by Fisher (1998). Neurobiological activity associated with sexual desire and arousal (Diamond and Dickenson, 2012; Cacioppo et al., 2012b) and oxytocin receptors (e.g., Bartels and Zeki, 2000; Acevedo et al., 2020) is consistently associated with romantic love. Fisher’s theory recognizes the role of sexual desire in romantic love (Fisher, 1998, 2000) but does not adequately recognize that activity of the attachment system (i.e., oxytocin; Schneiderman et al., 2012, 2014; Ulmer-Yaniv et al., 2016) may in fact constitute an aspect of romantic love. I, and others, believe a type of sexual desire (Bode and Kushnick, 2021; see also Diamond and Dickenson, 2012 for suggestions of different types of sexual desire) and the attachment system (Langeslag S. et al., 2012; Bode and Kushnick, 2021) form part of romantic love.

Fisher (1998) suggests that sexual desire is a feature of romantic love; she contends that one of the psychological properties associated with the attraction systems is “a sexual desire for the target of infatuation […]” (p. 32). In a later paper (Fisher, 2000), Fisher describes this sexual desire in people experiencing romantic love in the context of lust (sex drive). Sexual desire is associated with particular mechanisms and functions that differ from those said to be associated with the attraction system (Diamond, 2010; Diamond and Dickenson, 2012; Cacioppo et al., 2012a; Toates, 2014; Holloway and Wylie, 2015). Psychological evidence distinguishing sexual attraction from romantic attraction (Scheller et al., 2023) also supports the notion that sexual desire and attraction systems are separate, yet interdependent. Evidence that particular activation in the caudate tail, medial orbitofrontal cortex, right subcallosal cingulate, and right nucleus accumbens (NA) is predictive of relationship maintenance 40 months after being imaged while in love (Xu et al., 2012) suggests that these structures may play a particular role in pair bond formation.

Fisher’s (1998, 2000) and Fisher et al.’s (2002) narrow definition (and associated responsible mechanism) is inconsistent with the views of several others (e.g., Diamond and Dickenson, 2012; Cacioppo et al., 2012a; Langeslag S. et al., 2012; Bode and Kushnick, 2021). “[…Romantic] love is characterized by a subcortical AND a cortical brain network, in which each brain region might have a specific function” (Cacioppo et al., 2012a, p. 8). While Fisher’s theory explains some of what is associated with romantic love, it does not explain all of it (i.e., nerve growth factor, pair bond formation, similarities with mother-infant bonding, cortical structures associated with romantic love, or the desire for long term mating over short term mating). Sexual desire, as well as the attraction (which I divide into two types of attraction [courtship attraction and bonding attraction]) and attachment systems outlined by Fisher appear to work together so intricately and effectively in the early stages of a romantic relationship, their activity should be considered in terms of a single phenomenon - romantic love.

There appears to be more going on with romantic love than simply attraction and a focus of energies on a preferred mating partner. Factors not identified in Fisher’s (1998, 2000) and Fisher et al.’s (2002) model have been implicated in romantic love: cortisol (Marazziti and Canale, 2004; Weisman et al., 2015; Renner et al., 2021) and nerve growth factor (Emanuele et al., 2007). Opioids are also likely to play a role in romantic love (see Machin and Dunbar, 2011) but do not appear in Fisher’s model.

#### 3.3.3. The psychology of romantic love and early-stage parental love are similar

There is substantial overlap between the psychological characteristics of romantic love and those characterizing the early-stages of parental love (which includes mother-infant bonding). Leckman and Mayes (1999) provide a detailed description of the similarities between the early phase of romantic love and early-stage parental love. They find extreme similarities in the domains of altered mental state, longing for reciprocity, and establishment of intimate mutually satisfying reciprocal patterns of interaction usually marked by a culturally defined ritual. They also find substantial similarity in a range of other domains (i.e., exclusivity of focus, idealization of the other, heightened awareness of the other, heightened sense of responsibility, proximity-seeking, time-limited duration, need for things to be “just right,” and tendency to minimize altered patterns of thought and behaviors). The uncanny similarities between romantic love and parental love even extend to the use of “baby talk” between romantic lovers (Bombar and Littig, 1996). Leckman and Mayes (1999) highlight the similar trajectories of preoccupation in romantic love and parental love (discussed below).

There is some imprecision in the evidence of the psychological similarities between mother-infant bonding and romantic love. Leckman and Mayes (1999) chose to compare romantic love with early-stage parental love rather than early-stage maternal love. This makes it difficult to make specific inferences because the parental love of the father may have also evolved from mother-infant bonding mechanisms or pair bonding mechanisms and be a consequence of the evolution of pair bonding (see Lukas and Clutton-Brock, 2013; Opie et al., 2013). Regardless, parental love includes mother-infant bonding, as mother infant bonding is akin to maternal love, and therefore the argument that there are substantial similarities in the psychology of romantic love and mother-infant bonding holds true.

##### 3.3.3.1. Obsessive thinking is a discrete feature of romantic love and mother-infant bonding

Fisher (1998, 2000) and Fisher et al. (2002) speaks of obsessive thinking in people experiencing romantic love in terms of “intrusive thinking” and notes it is a hallmark of romantic love. This feature of preoccupation is reported regularly as a feature of romantic love (e.g., Hatfield and Sprecher, 1986; Langeslag S. J. E. et al., 2012; Brand et al., 2015). Mothers also experience obsessive thinking about their infants. Features of obsessive thinking in mothers of infants that have been measured empirically include having one’s daily routine interrupted by thoughts about their infant, thoughts about their infant interfere with concentration on other things, visually imagining their infant when not in direct contact, and thinking about their infant when at work or otherwise engaged (Kim et al., 2013). According to Leckman and Mayes (1999), in romantic love, preoccupation increases through the courtship phase and peaks at the point of reciprocity where preoccupation begins to slowly wane. In parental love, preoccupation increases throughout pregnancy and peaks at the point of birth where pre-occupation begins to slowly wane. I think there is probably more variability in the trajectory of preoccupation in these two states than suggested by the authors.

#### 3.3.4. The neurobiology and (neuro)endocrinology of romantic love and mother-infant bonding share similarities

##### 3.3.4.1. The neurobiology of romantic love and mother-infant bonding share similarities

The neurobiology of romantic love and maternal love have numerous similarities. Bartels and Zeki (2004) found that activity in several brain regions implicated in romantic love overlaps precisely with that involved in maternal love. This is particularly the case in regions associated with a high density of oxytocin and vasopressin receptors. Overlapping brain areas include regions in the striatum (i.e., putamen, globus pallidus, caudate nucleus), the middle insula, and the dorsal part of the anterior cingulate cortex. It should be noted that some of the mothers assessed in that study had children much older than infants. Nonetheless, this is consistent with endocrinological findings that higher levels of oxytocin are associated with the early stages of a romantic relationship (Schneiderman et al., 2012, 2014; Ulmer-Yaniv et al., 2016) and during maternal love, such as following mother–child contact (Feldman et al., 2010) and during pregnancy (Levine et al., 2007).

Neurobiological similarities between romantic and maternal love have been confirmed by two meta-analyses (Ortigue et al., 2010; Shih et al., 2022) which also highlighted distinct neural activity associated with each type of love (maternal love and romantic love). Ortigue et al. (2010), using a small number of studies, determined that romantic love was associated with “brain areas mediating emotion, motivation, reward, social cognition, attention, and self-representation” whereas maternal love “is mediated by a [periaqueductal (central) gray matter]-centered reward system, and also by higher-order cognitive or emotional cortical brain areas” (p. 3547–3548). Shih et al. (2022) who considered a much larger number of studies, found a more lateralized activity associated with maternal love than romantic love. Differences between maternal love and romantic love are interesting, and help to explain their different expression and functions, but are not evidence contradicting the theory of co-opting mother-infant bonding. Differences could be the result of the innovation that is a feature of co-option or as a result of changes over time to either maternal love, romantic love, or both. Importantly, both meta-analyses found substantial overlap in subcortical dopaminergic and oxytocinergic brain areas in different types of love (e.g., the [left] VTA; note Groppe et al., 2013 identified the VTA as the human brain site where oxytocin attaches salience to socially rewarding cues, although there is no evidence that oxytocin receptors are found in the primate VTA [see Zablocki-Thomas et al., 2022]).

Two reviews (de Boer et al., 2012; Cacioppo et al., 2012a) state that areas associated with emotional responses, dopamine rich reward pathways, and other areas active in romantic love overlap substantially with those found to be involved in maternal love (see Kikuchi and Noriuchi, 2015; Gholampour et al., 2020). Importantly, many of the recent studies that investigate maternal love involve, exclusively, mothers of children that were less than 9 months old (see Rigo et al., 2019; Shih et al., 2022) – the period in which mother-infant bonding is particularly important (see Bowlby, 1969/1982). This supports the notion that mother-infant bonding mechanisms were co-opted and restructured to serve their new function.

##### 3.3.4.2. Romantic love and mother-infant bonding involve activity of the dopamine system

As indicated above, dopamine-rich reward and motivation circuitry is implicated in both romantic love (Acevedo and Aron, 2014; Xu et al., 2015; Bode and Kushnick, 2021) and mother-infant bonding (Rigo et al., 2019). This largely explains psychological characteristics such as a desire for proximity and responsiveness to the loved one or infant. A genetic polymorphism that regulates dopamine 4 receptor density (DRD4-7R) is associated with both the maintenance of romantic love among newlyweds (Acevedo et al., 2020) and variation in maternal sensitivity to fussy infants (Kaitz et al., 2010). One study also found lower dopamine transporter density in lymphocytes in people experiencing romantic love than controls (Marazziti et al., 2017). This indicates an up-regulation of the dopamine system.

While substantial differences exist between romantic love and mother-infant bonding in some respects, such as the substantial lateralization of neural activity in mother-infant bonding (Rigo et al., 2019; Shih et al., 2022), the similarities are consistent with the theory of co-opting mother-infant bonding in the evolutionary history of romantic love. Activation of these areas is likely to have substantial impacts upon behavior above and beyond simply those associated with attraction and the focus of energies on a preferred romantic partner (e.g., affecting sleep and mood; see Bode and Kuula, 2021). Fisher (1998, 2000) and Fisher et al. (2002, 2006) contend that romantic love is the result of activation of a dopaminergic system. I contend that it is a necessary but not sufficient part of what causes romantic love.

The role of dopamine in romantic love, particularly the attraction systems, can best be described as playing an important role in selective reinforcement of association between the reward of social interaction and otherwise neutral stimuli (see Arias-Carrión et al., 2010 for a review of the dopaminergic reward system). It plays a particularly important role in learning and memory processes (Schultz, 2007) and motivates a type of “seeking” behavior (Alcaro et al., 2007). It does not appear to play a primary role in the pleasurable experiences associated with romantic love, something that may be better explained by activity of the opioid system (see Meier et al., 2021). It may, however, as Fisher (1998) suggests, play a role in the exhilaration, heightened energy, sleeplessness, and reduced appetite associated with romantic love.

##### 3.3.4.3. Romantic love and mother-infant bonding involve activity of the oxytocin system

Oxytocin is sometimes referred to as” the hormone of love” or the “love hormone” (see Carter, 2022; see also Carter, 2017a for review of oxytocin, including in relation to love). Findings indicate that higher levels of oxytocin are associated with the early stages of a romantic relationship (Schneiderman et al., 2012, 2014; Ulmer-Yaniv et al., 2016) and during maternal love, such as following mother–child contact (Scatliffe et al., 2019) and during pregnancy (Levine et al., 2007). A recent systematic review found substantial evidence for oxytocin’s role in human parenting behaviors and parent–child bonding (Shorey et al., 2023).

Oxytocin plays a role in maternal behavior as well as mate preferences and pair bonding in rodents (Froemke and Young, 2021). Recently, however, it has been demonstrated that oxytocin receptor-mediated signaling is not necessary for social attachment, parturition, and parental behavior in prairie voles (Berendzen et al., 2022), raising interesting questions about the causal mechanisms of these behaviors. The oxytocin system is thought to be a driving system in maternal and parental behavior (see Numan, 2020). However, it is interesting to note that circulating oxytocin levels are greater in people in the early stages of a romantic relationship than in people who have recently become parents (Ulmer-Yaniv et al., 2016).

Much emphasis has been placed on activity of the mesolimbic dopamine pathway in romantic love (e.g., Acevedo and Aron, 2014; Xu et al., 2015; Bode and Kushnick, 2021). This pathway is also active in mother-infant bonding (Rigo et al., 2019). Structures specifically implicated in both romantic love and mother-infant bonding include the VTA, NA, and amygdala, with particularly notable similarities in the left VTA (see Shih et al., 2022). These structures, while certainly rich in dopamine receptors, and which play a role in an identifiable reward and motivation system, are also mediated by other types of receptors, including oxytocin receptors (Baskerville and Douglas, 2010). Oxytocin activity interacts with the dopamine system and plays a role in social learning, memory, and motivation, especially towards sexual behavior and pair bond formation (see Baskerville and Douglas, 2010). In fact, social reward requires activation of pre-synaptic oxytocin receptors in the NA in mice (see Dölen et al., 2013). Evidence from rodents suggest that activation of oxytocin receptors in the VTA is probably critical for the rewarding and reinforcing properties of social interaction (see Hung et al., 2017; Borland et al., 2018; Xiao et al., 2018). This suggests that oxytocin plays an important role in attraction, not just attachment.

Other animal studies also indicate that oxytocin modulates a number of brain circuits involved in cognition, many of which are implicated in maternal care (see Mitre et al., 2017), and, which I also suggest, are implicated in romantic love. These include processing of sensory stimuli, social recognition, social memory, and fear. The relationships between dopamine and oxytocin systems (detailed below) lend support to the notion that oxytocin acts on motivation pathways by increasing the salience of specific social stimuli (Froemke and Young, 2021) which results in up-regulation of dopamine pathways (see Love, 2014). Oxytocin is also associated with neural plasticity in rodents (Froemke and Young, 2021), and this may account for the increased nerve growth factor activity in people experiencing romantic love (see Emanuele et al., 2006; see also Luppi et al., 1993).

While oxytocin has been referred to as the “love hormone” (see Carter, 2022 abstract for authority), this seems to be misguided. Increasingly, evidence of the primacy of the opioid system in strong social attachments (see Panksepp, 2005; Machin and Dunbar, 2011) and the evidence that oxytocin receptors are not necessary for bonding in prairie voles (Berendzen et al., 2022) suggests that opioids may in fact be the more appropriate candidate for such a name (see Panksepp, 2005; Machin and Dunbar, 2011 for critique of the oxytocin “love hormone” claim).

##### 3.3.4.4. Romantic love and mother-infant bonding involve activity of the endogenous opioid system

Machin (2022) contends that the endogenous opioid system (i.e., *β*-endorphin) is the common mechanism among the different types of love (e.g., maternal love, parental love, romantic love, companionate love, familial love, platonic love, brand love, love of pets, love for a celebrity, love of country, love of a god). This is based on the BOTSA (introduced above) and supported by research demonstrating that endogenous opioids play an important role in the full breadth of close social relationships in non-primate mammals, non-human primates, and humans (see Machin and Dunbar, 2011). There is a breadth of evidence demonstrating opioid activity in mother-infant and sexual/romantic interactions in a range of species (see Machin and Dunbar, 2011 for summary) and in social monogamy behaviors (i.e., pair bonding and social attachment) in non-human primates (see French et al., 2018 for summary). The opioid system (i.e., the mu receptor) has been implicated in pair bond formation in monogamous prairie voles (see Loth and Donaldson, 2021). There is also evidence that circulating *β*-endorphin levels are higher in individuals in the early stages of a romantic relationship and recent parents following interactions with their partner or infant (Ulmer-Yaniv et al., 2016).

Endorphins are involved in bonding and reproduction in mammals in multiple ways. Endorphin receptors and transmission is found throughout the mesolimbic pathway (i.e., VTA, NA, amygdala) and have been associated with reproductive behaviors in rats (McGregor and Herbert, 1992; van Furth and van Ree, 1996; Olive et al., 2001). The opioid system has additional downstream impacts on mesolimbic pathway activity (see Pierce and Kumaresan, 2006). For example, withdrawal from opiates in rats can result in a down-regulation of the mesolimbic system which can persist long after the somatic symptoms of withdrawal end (Diana et al., 1999). This type of effect may account for the substantial mesolimbic activity associated with romantic relationship dissolution (see van der Watt et al., 2021; see also Bales and Rogers, 2022 for discussion of Κ opioids in partner loss). This suggests that romantic love may be similar to opioid addiction consistent with Fisher et al. (2016) suggestion about cocaine and amphetamine addiction (Fisher et al., 2016; see also Bode and Kushnick, 2021 for description of similarities between romantic love and addiction). The opioid system has been implicated in mother-infant social motivation and bonding in rodents (see Panksepp et al., 1994) and opioid stimulation in the VTA is associated with the onset of maternal behaviors in rats (Thompson and Kristal, 1996).

## 4. Romantic love requires activation of the bonding attraction and attachment systems

Much of the evidence provided above does not indicate that the theory of co-opting mother infant bonding is correct. It details substantial similarities between romantic love and mother-infant bonding. It also shows that the attachment system in humans (characterized by oxytocin, and probably, vasopressin activity [Carter, 2017a, 2017b], as well as involving other systems such as opioids, serotonin, and dopamine) is heavily associated with romantic love. It does not, however, confirm that it is a feature of romantic love rather than simply the activity of a closely intertwined system, as Fisher (1998, 2000, 2016) and Fisher et al. (2002, 2016) contend. In this section, with some degree of support, I move beyond mere speculation to suggest that the state of romantic love necessarily involves the attachment system.

I re-assert that romantic love serves a pair bonding function (Fletcher et al., 2015; Bode and Kushnick, 2021). Distinct pair bond formation and pair bond maintenance functions and mechanisms exist in animals (see Loth and Donaldson, 2021; Duclot et al., 2022), and there is every reason to believe that this is the case in humans (see Sprecher et al., 2008; Ogolsky and Monk, 2019; Ogolsky and Stafford, 2022 for conceptually related work in humans). Romantic love, specifically, serves a pair bond formation function. The attachment system plays a role in both pair bond formation and pair bond maintenance. I also believe the attraction system plays a role in pair bond formation. The evidence I draw on comes from animal models (i.e., monogamous prairie voles) but I also draw on the available human evidence. It supports, to some degree, the notion that both the attraction and attachment systems are active in romantic love.

### 4.1. The basic premise

The basic premise is that throughout a period in which an individual is experiencing romantic love, the attraction, attachment, and obsessive thinking systems are active. All three systems appear to have been co-opted in romantic love. Dopamine-oxytocin interactions serve to instigate and promote attraction, attachment, and pair bonding (i.e., pair bond formation). In circumstances of reciprocated romantic love and well-functioning relationships (i.e., when regular interaction, proximity, physical touch, and verbal exchange are common), mechanisms of romantic love ramp up activity of the attachment system. In circumstances where such stimuli are not present (i.e., in some cases of unrequited love), this process is still occurring (possibly facilitated by obsessive thoughts), but does not progress to the formation of attachment, full activation of the attachment system, and transition to pair bond maintenance. This explains why, in circumstances of fast-arising romantic love (or in any type of romantic love), the adaptive nature of mate choice may give way to some of the maladaptive features of infatuation (i.e., physical instability, loss of appetite, targeted social anxiety, clammy hands, physical tension, sleep difficulties, shyness; see Langeslag S. et al., 2012). Some of these sequelae are analogous to the symptoms of acute cocaine intoxication demonstrating a potential role for dopamine in these experiences (see Gay, 1982). These features of infatuation may be more common when the dopaminergic activity of mate choice mechanisms (i.e., attraction) are active without substantial calmative effect of the oxytocinergic attachment system (see Marazziti et al., 2021).

The infatuation component of romantic love may have resulted from a co-opted bonding attraction system (i.e., the left VTA; see Shih et al., 2022) merged with a pre-existing courtship attraction system, while the attachment component results from the co-opted attachment system. Both systems play a role in pair bond formation. Whereas Fisher et al. (2016) and others (e.g., Marazziti et al., 2021) believe that romantic love precedes a period of pair bonding, I assert that part of romantic love is the process of pair bonding (i.e., pair bond formation).

The proposition that romantic love involves activity of both the attraction and attachments systems differs with the views of others. Marazziti et al. (2021) describe a process in which oxytocin is produced in the hypothalamus (one of the regions where attraction is generated) and that transforms anxiety/fear reactions into a sense of “well-being, reward, and joy” (p. 252). They contend that this may be because of activation of dopaminergic reward processing and they imply that oxytocin may play a direct role in this downstream activation. They couch this interaction in terms of two distinct steps of “love” and suggest that this is the result of the activation of reward processing by dopamine. A recent systematic review of functional neuroimaging of the human hypothalamus in socioemotional behavior recognizes the hypothalamus’ role in romantic love and pair bonding (Caria and Dall'Ò, 2022).

Unlike the model proposed by Fisher (1998, 2000), Fisher et al. (2002), and Marazziti et al. (2021), the theory of co-opting mother-infant bonding suggests that oxytocinergic activity is a necessary component of romantic love, and the “second step” that Marazziti et al. (2021) refer to is, in fact, part of romantic love. It may very well be that multiple forms of love assume activity of the oxytocin-heavy attachment system. Just as Machin (2022) contends that one common factor among all types of love is the endogenous opioid system, the broader attachment system may be another more generalized common factor in some (or all) types of love (See Ortigue et al., 2010). Love, as understood in the English language, may simply be the psychological expression of attachment or bonds. This would mean that romantic love, by definition, requires activity of both the bonding attraction system (likely to be responsible for the romantic characteristics of romantic love – mate choice, courtship, pair bond formation), and attachment system (likely to be responsible for the love component of romantic love – pair bond formation).

### 4.2. Evidence that romantic love involves activity of the bonding attraction and attachments systems

This subsection outlines the animal and human evidence that romantic love involves activation of both the bonding attraction and attachments systems. The animal evidence I draw on below is summarized in a review by Loth and Donaldson (2021) on oxytocin, dopamine, and opioid interactions underlying pair bonding, although others have addressed these issues (e.g., Walum and Young, 2018). The attachment system is primarily associated with oxytocin and vasopressin (Fisher, 1998; Carter, 2017a, 2017b), but involves dopamine and opioids (peptides consistently implicated in pair bond formation; see Numan and Young, 2016; Walum and Young, 2018; Loth and Donaldson, 2021). Key to supporting the claim that romantic love necessarily involves activation of the attachment system (and, in turn, supporting the theory co-opting mother-infant bonding in the evolutionary history of romantic love), is dopamine-oxytocin interactions in pair bond formation. The human evidence supporting the notion that romantic love necessarily involves activation of the bonding attachment system is not substantial, and in fact partly relies on the assumption that romantic love does play a pair bond formation function (see Fletcher et al., 2015; Bode and Kushnick, 2021). It also relies on circumstantial evidence of particular structures associated with romantic love playing a role in pair bond formation (see Xu et al., 2012 discussed below). However, in conjunction with the animal evidence of the dopamine-oxytocin interactions in pair bond formation (see below), this limited human evidence does set the scene for future research to test the hypothesis.

#### 4.2.1. Key dopamine-oxytocin interactions during pair bond formation in prairie voles

Individual neurotransmitter systems are intricately intertwined with other neurotransmitter systems. This is certainly the case with the oxytocin system. Recent reviews have outlined its interaction with the opioid system in regulating social behavior (Putnam and Chang, 2022), dopamine and serotonin systems in regulating different components of motherhood (Grieb and Lonstein, 2022), and a range of neuromodulators in a number of complex social behaviors such as social learning and maternal behavior (Paletta et al., 2022). These are all relevant to the theory of co-opting mother infant bonding in the evolutionary history of romantic love. However, in light of the theory of independent emotion systems (Fisher, 1998, 2000; Fisher et al., 2002) and its emphasis on dopaminergic structures (e.g., Fisher, 2006), what is most relevant here are dopamine-oxytocin interactions that play a role in pair bond formation.

During mating, in rodents, oxytocin is released in the amygdala, hippocampus, and VTA which directly stimulates the mesolimbic dopamine pathway projecting to the NA and the prefrontal cortex (Baskerville and Douglas, 2010). Interestingly, stimulation of either central dopamine or central oxytocin in rodents causes similar social and affiliative behaviors, including sexual behavior (see Baskerville and Douglas, 2008) and pair bond formation (i.e., in monogamous prairie voles; see Wang and Aragona, 2004; see also Liu and Wang, 2003; Walum and Young, 2018; Loth and Donaldson, 2021 for additional consideration of dopamine-oxytocin interaction in pair bond formation). However, it has been demonstrated that concurrent oxytocin (i.e., to OXTR) and dopamine (i.e., to D2R) signaling is required for pair bond formation in female prairie voles (Liu and Wang, 2003). Importantly, neither of these systems serves as an upstream regulator of the other during bond formation. Oxytocin receptors in monogamous prairie voles have a greater density in the NA than in polygynous voles (Ross et al., 2009) and blocking oxytocin receptors in the NA prevents partner preferences (see Young et al., 2001). These receptors appear to play a particular role in affiliative behaviors (see Insel, 2010). Oxytocin may also interact with dopamine by facilitating synaptic plasticity to link neural representations of partner cues to the mesolimbic pathway (Walum and Young, 2018).

#### 4.2.2. Human evidence

##### 4.2.2.1. Evidence of pair bond formation structures

As indicated above, Xu et al. (2012) found that greater activation in the caudate tail and less activation in the medial orbitofrontal cortex, right subcallosal cingulate, and right NA in people who were experiencing romantic love predicted relationship maintenance 40 months later. This suggests a role for these structures in pair bond formation. Fisher et al. (2005) contend that the neural mechanism for mate choice (i.e., attraction) involves multiple reward regions, and these regions constitute the mechanisms of the attraction system. The medial orbitofrontal cortex (see Schultz, 2001) and right NA (see Salgado and Kaplitt, 2015) most definitely play roles in reward processing. The subcallosal cingulate, however, tends to be more associated with emotion and mood (see for example Mayberg et al., 1999). The role of the caudate tail in romantic love and pair bond formation is even more unclear, as it tends to be associated with the processing of visual information, movement control (Graff-Radford et al., 2017) and learning acquisition (Seger and Cincotta, 2005), although the head of the caudate is associated with processing reward-related information (Delgado et al., 2000). It is possible that activity of the caudate tail represents the shift in social cognitive functions (i.e., social recognition of mate choice and pair bonding) from olfaction to visualization in primates compared to rodents (see Broad et al., 2006; Kavaliers and Choleris, 2017; Walum and Young, 2018).

The medial orbitofrontal cortex has been implicated in romantic love using multiple methods (e.g., Xu et al., 2011; Takahashi et al., 2015) and, importantly, has been specifically implicated in long-term romantic love (Acevedo et al., 2012) and long-term committed relationships, generally (Ueda and Abe, 2021). This suggests a role of the medial orbitofrontal cortex in attachment. The medial orbitofrontal cortex includes a large number of dopamine receptors and intranasal oxytocin administration is associated with greater orbitofrontal activation in response to touch (Chen et al., 2020), suggesting some degree of dopamine-oxytocin interaction that may mediate the influence of interpersonal inputs on pair bond formation. Intranasal oxytocin administration has also been shown to promote self-interested behaviors which was associated with greater medial orbitofrontal cortex activation (Xu et al., 2019). This may suggest the medial orbitofrontal cortex plays a role in focusing efforts on a loved one at the expense of others, promoting pair bond formation. The NA has been implicated in established romantic relationships and can distinguish between a long-term romantic partner and an attractive face (Ueda and Abe, 2021). This suggests that the NA probably plays a role in attachment, as well as attraction. Animal studies highlight the necessary role of oxytocin (and serotonin) receptors in the NA in processing reward (e.g., Dölen et al., 2013) and the role of oxytocin in the NA in maternal attachment and pair bonding has been emphasized (Dölen and Malenka, 2014). In particular, differences in size have been identified between the left and right NA (Ahsan et al., 2007) although findings have been inconsistent (see Salgado and Kaplitt, 2015 for a comprehensive review of the NA). This may indicate functional lateralization relevant to pair bond formation. The role that the caudate tail and subcallosal cingulate, may play in human pair bond formation and activity of the attachment system is yet to be elucidated and should be the target of future research.

##### 4.2.2.2. Oxytocin activity is associated with the early stages of romantic love

One other line of evidence that romantic love involves activation of the attachment system comes from increased circulating oxytocin levels in individuals in the early stages of a romantic relationship. Unfortunately, none of the studies that I refer to here used validated measures of romantic love. Nonetheless, inferences can be made about romantic love because this is the period in which romantic love often manifests. The sample in the first study to measure circulating oxytocin (i.e., in plasma; Schneiderman et al., 2012) found significantly higher levels of oxytocin following dyadic interactions in a group of individuals who were in a romantic relationship from between 2 weeks and 4 months (mean = 2.4 months) compared to singles. The second study to measure circulating oxytocin (i.e., in serum; Schneiderman et al., 2014) found significantly higher levels of oxytocin following conflict interaction in a group of individuals who were in a romantic relationship between 1.5 and 3 months (mean = 2.4 months) compared to singles. The third study to investigate circulating oxytocin levels (i.e., in plasma; Ulmer-Yaniv et al., 2016) found significantly higher levels of oxytocin following in dyadic interaction in a group of individuals who were in a romantic relationship from between 3 weeks and 4 months compared to singles. The fact that oxytocin levels were elevated in individuals during the very early stages of a romantic relationship suggest that the early stages of romantic love may involve activity of the oxytocin-heavy attachment system. This serves to promote pair bond formation by, in part, reducing interest in others (Freeman et al., 2021).

##### 4.2.2.3. Evidence of additional attachment system structures

Acevedo and Aron (2014) provide a concise account of the neural correlates of pair bonding over time. They emphasize that differences in activation of the ventral putamen/ pallidum, anterior cingulate, some areas of the NA, and periaqueductal gray relate to length of time in a relationship. Aron et al. (2005) specifically found that relationship length was associated with specific activation in the right mid-insular cortex; the right anterior and posterior cingulate cortex; and the right posterior cingulate/retrosplenial cortex as well as the left inferior frontal gyrus, left middle temporal gyrus, left ventral putamen pallidum, and left posterior cingulate/retrosplenial cortex. Structures and systems that interact with these regions may play a particular role in pair bond formation and the ramping up of the attachment system. However, it also seems that activity in these regions represents a transition from pair bond formation to pair bond maintenance.

### 4.3. Pair bonding involves two distinct phases with distinct functions and mechanisms

Pair bonding involves two distinct phases with distinct functions and mechanisms (i.e., pair bond formation and maintenance). In the prairie vole, which serves as the predominate animal model for pair bonding in humans, the research clearly distinguishes between pair bond formation and pair bond maintenance. Pair bond formation is the period in which a pair bond is created and is associated with mating. In prairie voles, pair bond formation is hypothesized to involve two distinct plasticity processes: “the formation of a distinct neural representation of the partner, allowing for partner recognition, and a persistent attraction to the partner that continues after mating, leading to a partner preference” (Walum and Young, 2018, p. 6). The mechanisms most notably associated with pair bond formation in prairie voles are driven by oxytocin, dopamine, and opioid systems (see Numan and Young, 2016; Walum and Young, 2018; Loth and Donaldson, 2021). The primary mechanisms involve oxytocin and dopamine 2 receptors in the NA (at least in females) in relation to dopamine-oxytocin interactions, oxytocin and mu opioid receptors in the NA in relation to oxytocin-opioid interactions, and dopamine 2 and mu opioid receptors in the NA shell and striatum in relation to dopamine-opioid interactions (see Loth and Donaldson, 2021 for informative summary).

Pair bond maintenance is the period following that in which a pair bond has been created. Behaviors associated with pair bond maintenance in the prairie vole include a preference for a partner and aggression towards potential alternative mates of a partner (see Lee et al., 2019). The mechanisms most notably associated with pair bond maintenance in prairie voles are also driven by oxytocin, dopamine, and opioid systems (see Numan and Young, 2016; Walum and Young, 2018; Loth and Donaldson, 2021); however, these mechanisms may differ from those found in pair bond formation. The primary mechanisms likely involve oxytocin, dopamine 1, and dopamine 2 receptors in the NA and/or the medial prefrontal cortex in relation to dopamine-oxytocin interactions, oxytocin and kappa opioid receptors in the NA in relation to oxytocin-opioid interactions, and dopamine 1 and kappa opioid receptors in the NA shell (at least in males) in relation to dopamine-opioid interactions (see Loth and Donaldson, 2021 for informative summary). This phase represents established pair bonds. What is clear is that the mechanisms associated with pair bond formation and maintenance in prairie voles differ, but both involve mechanisms associated with the attachment system. That pair bonding involves two distinct phases aligns with the fact that maternal love involves two distinct phases: mother-infant bonding (i.e., at least 9 months where the bonding is almost entirely maternal-driven; see Bowlby, 1969/1982) and a later period of further love, where attachment behaviors are exhibited by the child.

#### 4.3.1. The traditional approach to love in adult romantic relationships aligns with distinct phases of pair bonding

Seminal conceptions of love proposed by Elaine Hatfield (e.g., Berscheid and Walster, 1969; Walster and Walster, 1978; Hatfield and Rapson, 1993) differentiated passionate love from companionate love. Passionate love (referred to in this article as “romantic love”) was said to be “[a] state of intense longing for union with another. Passionate love is a complex functional whole including appraisals or appreciations, subjective feelings, expressions, patterned physiological processes, action tendencies, and instrumental behaviors. Reciprocated love (union with the other) is associated with fulfillment and ecstasy; unrequited love (separation) with emptiness, anxiety, or despair” (Hatfield and Rapson, 1993, p. 5). Companionate love, on the other hand, is felt less intensely, often follows a period of romantic love (Hatfield and Walster, 1985), and merges feelings of intimacy and commitment (Sternberg, 1986). In my assessment, passionate love aligns with the pair bond formation phase of pair bonding and companionate love aligns with the pair bond maintenance phase.

## 5. A new evolutionary approach

**Figure 1:**
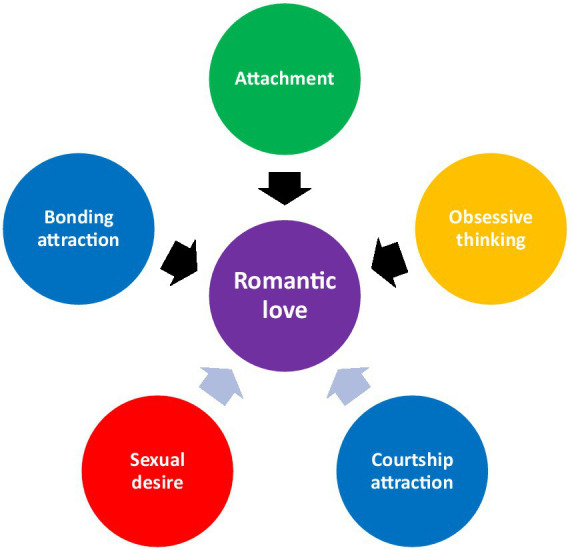
Five systems that contribute to romantic love. Black arrows represent co-option.

The theory of co-opting mother-infant bonding outlines two evolutionary processes whereby distinct neurobiological and (neuro)endocrinological systems were merged into a single phenomenon (romantic love) to create a variety of psychological outcomes. Figure 1 presents the two evolutionary processes through which romantic love was formed (i.e., co-option and combination) and the mechanisms involved.

### 5.1. Co-opted components

Mother-infant bonding involves three components: bonding attraction, attachment, and obsessive thinking. Each component has its own mechanisms and psychological outputs, and these appear to have been co-opted in romantic love. Mother-infant attraction is associated with dopamine and oxytocin activity (most notably characterized by activity in the left ventral tegmental area (VTA), right thalamus, left substantia nigra, and putamen (Shih et al., 2022), among other areas (see Rigo et al., 2019). It may be associated with psychological features like proximity-seeking, physical touch, exclusivity of focus, heightened awareness of the loved one, cognitive biases, and possibly, the loved one taking on a special meaning. Evidence of co-option is strongest in relation to activity of the left VTA in romantic love (Shih et al., 2022).

Mother-infant attachment is associated with dopamine, oxytocin, and opioid activity (see Numan and Young, 2016; Walum and Young, 2018) and most notably characterized by increased oxytocin following contact between the mother and infant (Scatliffe et al., 2019). Animal evidence (see Carter, 2017a, 2017b) also suggests that vasopressin probably also plays a role, although the human evidence is limited (see Bartels and Zeki, 2000; Acevedo et al., 2020). There is also evidence of higher levels of circulating *β*-endorphin (an opioid) in early-stage mothers following contact with their infant (Ulmer-Yaniv et al., 2016). Attachment may be associated with psychological features like heightened sense of responsibility, longing for reciprocity, a powerful sense of empathy towards the infant, and, perhaps, a sense of love. The thalamus plays an important role in attachment (see Marazziti et al., 2014). Evidence of co-option is strongest in relation to the increased circulating oxytocin measured in people in the early stages of a romantic relationship following contact (Schneiderman et al., 2012, 2014; Ulmer-Yaniv et al., 2016).

Obsessive thinking is most characterized in mother-infant bonding and romantic love by obsessive thoughts about the infant/ loved one (e.g., thoughts about an infant/ loved one interfere with concentration on other things; see (Leckman and Mayes, 1999; Bajoghli et al., 2013; Kim et al., 2013). Little is known about the mechanisms that are associated with obsessive thoughts during mother-infant bonding. While some have speculated that obsessive thought associated with preoccupation in romantic love is the result of a down-regulated serotonin system (e.g., Fisher, 1998, 2000; Fisher et al., 2002, the evidence suggests otherwise (see Bode and Kushnick, 2021). Others have suggested that it may involve the anterior cingulate (Aron et al., 2005), parts of which are densely populated with serotonin receptors (Palomero-Gallagher et al., 2009), or that oxytocin may play a role in maternal cognition and behavior indicative of preoccupation (see Leckman et al., 1994). Most of this speculation relies on the belief that obsessive thinking associated with romantic love share some mechanistic similarities with intrusive thoughts found in obsessive compulsive disorder (see Leckman and Mayes, 1999). This is possible but does not seem certain to me as these two types of thoughts differ in both function and content. Evidence for co-option of obsessive thinking comes from the marked and unique similarities between mother-infant bonding and romantic love.

### 5.2. Combined components

The theory of co-opting mother-infant bonding in the evolutionary history of romantic love also indicates that two other systems were combined with co-opted components and modified in romantic love: courtship attraction and sexual desire. Courtship attraction is associated with dopamine and oxytocin activity (possibly characterized by bilateral activation of the VTA; Shih et al., 2022), among other factors. It is associated with mate choice, focusing energies on a preferred mating partner, and courtship efforts. It can take the form of crushes or acute courtship attraction episodes. Evidence of modification from an original form comes from the fact that a courtship attraction system, in most of our evolutionary history, would have been tailored towards short-term (promiscuous) mating (see Buss and Schmitt, 1993; Schacht and Kramer, 2019), but now can facilitate long-term mate choice in most people.

Sexual desire, as it relates to romantic love, may be associated with testosterone (and other factors), and is characterized by activity in the caudate, insula, putamen, and anterior cingulate cortex (Diamond and Dickenson, 2012). Testosterone, along with dopamine, serotonin, norepinephrine, acetylcholine, histamine, opioids have all been implicated in sexual behavior (Calabrò et al., 2019).

Sexual desire would be associated with both proceptivity (a drive to initiate sexual activity) and receptivity of sexual activity in both sexes (see Baum et al., 1977; Meston and Buss, 2007, 2009; Diamond, 2010). Evidence of modification from an original form comes from the fact that sexual desire among people experiencing romantic love is more persistent and intense than in individuals not experiencing romantic love. Sexual activity in people experiencing romantic love is greater than among couples who are not experiencing romantic love. There is also some suggestion from a meta-analysis of sexual desire and three types of love that the activity of the insula may differ between sexual desire and love (Cacioppo et al., 2012a, 2012b).

It should be noted that, while bonding attraction, attachment, and possibly, obsessive thinking are necessary components of romantic love, courtship attraction and sexual desire are not. Individuals who fall in love with a romantic partner some time after romantic relationship formation may not experience the courtship attraction that can precede a romantic relationship. Some, (e.g., Diamond, 2003) have also noted that romantic love can occur in the absence of sexual desire. As such, bonding attraction, attachment, and obsessive thinking may be considered the core of romantic love, and courtship attraction and sexual desire may be considered causally linked adjuncts.

## 6. Unanswered questions in the evolutionary history of romantic love

A theory of co-opting mother-infant bonding accounts for parts of the evolutionary history of romantic love. This theory suggests that some of the mechanisms needed for romantic love existed before its emergence in the form of mother-infant bonding mechanisms. However, there are several questions that remain to be answered before an understanding of the evolutionary history of romantic love can be postulated. These include important questions around the relationship between the emergence of romantic love and the evolution of pair bonding as well as the precise timeframes in which pair bonding evolved.

### 6.1. Did romantic love evolve in the context of pair bonding?

Fisher (see Fisher, 2016; Fisher et al., 2016) and others (Bode and Kushnick, 2021) suggest that romantic love evolved in the context of pair bonding. This hypothesis, however, is not sufficiently developed to paint a clear picture of the evolutionary history of romantic love. There is some degree of imprecision in Fisher’s (2016) and Fisher et al.’s (2016) argument and Bode and Kushnick (2021) rely almost entirely on the premise that romantic love serves a pair bond formation function to make their argument.

Fisher (1998, 2000) and Fisher et al. (2002) contend that the attraction system evolved independently of the sex drive and attachment systems and claims that these systems became increasingly separate from each other over time (Fisher, 1998). However, this seems, on the face of it, inconsistent with the claim that “the neural structures associated with feelings of intense romantic love and partner attachment evolved in conjunction with the evolution of the human predisposition for pair-bonding” (Fisher et al., 2016, p. 4). Fisher is also unclear in terms of pinpointing when romantic love emerged versus when it changed through a continuous process of evolution.

Bode and Kushnick (2021) do not provide any evidence for their claim that romantic love evolved in the context of pair bonding but simply quote Walum and Young (2018): “[…] pair bonding is the evolutionary antecedent of romantic love and […] the pair bond is an essential element of romantic love” (p. 12). While there seems to be some agreement that romantic love evolved in the context of pair bonding, this link needs clearer articulation in an internally consistent manner that draws on evidence. Bode and Kushnick’s (2021) definition of romantic love states that one of the functions of romantic love is pair bonding. To that extent, they can be afforded liberty to claim that it only evolved in the context of the evolution of pair bonding. However, they also fail to differentiate between when an antecedent to romantic love emerged and the period in which it changed through a continuous process of evolution. It is, therefore, necessary to hypothesize what form this relationship took in the environment of evolutionary adaptedness (Bowlby, 1969/1982; see also Cosmides and Tooby, 1987) and future work should attempt to do this.

The sum of evidence is not entirely convincing that an initial antecedent to romantic love first emerged in the context of pair bonds (although it is feasible). I think that it is wise to consider the possibility, first proposed by Fisher et al. (2016) in *Anatomy of Love*, that an antecedent to romantic love emerged prior to the emergence of pair bonds, in the form of a type of seasonal pair bond that lasted only one reproductive cycle (such as is the case in some birds; see Emery et al., 2007). This would draw into question the claim that “[…] pair bonding is the evolutionary antecedent of romantic love […]” (Walum and Young, 2018, p. 12). This would also suggest that the emergent antecedent to romantic love was in fact, the original antecedent to pair bonds. Such a state could have ensured a male could provide for a female while pregnant or until the mother stopped breastfeeding and could potentially fall pregnant again. Fisher et al. (2016) contends this could have been about a 3-to-4 year bond. Given that altriciality (i.e., infant helplessness) in humans is probably more recent than the proposed emergence of the antecedent to romantic love (Rosenberg, 2021), I suspect it was initially for a shorter period than this. Such a bond would also explain why short-term serial monogamy (see Fisher, 2012 for explanation of serial monogamy) appears to be a common mating strategy among many humans, especially young adults. There is no doubt, however, that romantic love changed and evolved over time in the context of the evolution of pair-bonds, because modern romantic love serves a pair bond formation function.

### 6.2. When did pair bonding evolve?

The timing of the emergence and evolution of pair bonding is uncertain. Fisher et al. (2016) contend that pair bonding could have evolved at any point in hominin history (see Wood and Lonergan, 2008). Bode and Kushnick (2021) propose four hypotheses about the emergence of pair bonding. One hypothesis places its emergence prior to the hominin line split from the last common ancestor with chimpanzees and bonobos (approximately 8–5 million years ago; see Almécija, 2016). Three of their hypotheses, however, place its emergence in line with Fisher et al.’s (2016) postulation. Fisher (2000, 2016) and Fisher et al. (2016) suggest that a likely timeframe for the evolution of monogamy (and therefore pair bonding and romantic love) was more than 4 million years ago in conjunction with bipedalism, hominin adaptation to a woodland/savannah eco-niche, and the need for females to carry infants in their arms.

Fisher et al. (2016) draw on work by Lovejoy (2009) which suggests that *Ardipithicus ramidus*, which lived approximately 4.4 million years ago, possessed a number of morphological characteristics indicative of monogamy and pair bonding (i.e., bipedality, loss of honing canine, and ovulatory crypsis). Therefore, they suggest, romantic love, which evolved “in conjunction” with pair bonding, may have evolved around the time of that species. Fisher et al. (2016) also suggest that *Australopithecus afarensis*, dated to about 3.5 million years ago, was sexually dimorphic in a way similar to modern humans. Low sexual dimorphism is indicative of monogamy, and therefore, pair bonding, in mammals (Kleiman, 1977). It is also associated with a reduction in intrasexual physical competition in primates (see Leutenegger and Kelly, 1977) which is indicative of human pair bonding and monogamy.

There is now some convincing suggestion that bipedalism may have originally emerged long before 4.4. million years ago (see Kivell and Schmitt, 2009; Böhme et al., 2019) indicating that this selective pressure could have resulted in the emergence of pair bonding behaviors prior to the hominin line split from the common ancestor with chimpanzees and bonobos. This would be consistent with hypothesis 1 from Bode and Kushnick (2021). If the timeframe for the emergence of pair bonding advanced by Fisher et al. (2016) is correct, a more likely selective pressure is the evolution of altriciality and large brain size at birth (see Rosenberg, 2021), which occurred around the base of the genus *Homo* about 2 million years ago in *Homo ergaster* and *Homo erectus*. Some postulate altriciality to have emerged 3–4 million years ago (see Rosenberg, 2021 for review of the fossil record; see also Halcrow et al., 2020). Additionally, some comprehensive work indicates hominin species more proximal to modern humans (e.g., *Homo erectus*, *Homo floriensis*, *Paranthropus boisei*, *Paranthropus robustus*) were much more sexually dimorphic than modern humans (i.e., see Grabowski et al., 2015). While there have been suggestions, cited by Fisher et al. (2016), that archaic hominins (i.e., *Australopithecus afarensis*) were sexually dimorphic in body mass to a similar degree to modern humans (Reno et al., 2003), others (Plavcan et al., 2005; Gordon et al., 2008) have discounted this possibility (see Schacht and Kramer, 2019 for succinct summary of some of the evidence for sexual dimorphism in hominin history). In fact, it is suggested that much of the *Homo* and archaic hominin lineage possessed a male to female sexual dimorphism ratio in body mass in the range of 1.2–1.6, notably greater than the 1.1 characteristic of *Homo sapiens* (Grabowski et al., 2015).

The presence of characteristics indicative of monogamy and pair bonding in our distant ancestors but body mass sexual dimorphism only reaching current levels by at least about 500,000 years ago (see Carretero et al., 2012; see also García-Campos et al., 2020 for dental evidence) suggests that there may have been a number of steps in the evolution of pair bonding (i.e., social monogamy and bonded polygyny) from promiscuous or harem-based polygynous systems (see Schacht and Kramer, 2019). It is also important to distinguish between the emergence of antecedent states, processes, functions, and mechanisms, and the entire process of evolution (which Fisher does in *Anatomy of Love*; Fisher, 2016). I concur with Fisher (2016) that pair bonding, like that expressed by modern humans, is likely to have evolved in a period extending to much later than that proposed by Fisher et al. (2016), although its antecedents may have emerged by at least in the vicinity of the timeframe they propose.

## 7. Areas for future research

The case for the co-opting of mother-infant bonding in the evolution of romantic love highlights the need for further and more precise research. One of the limitations of Fisher’s (1998, 2000) and Fisher et al.’s (2002) model is that it has not generated the widescale hypotheses and research that would normally be expected of a model such as hers. Part of the reason for this may be that her model, as it relates to romantic love being constituted entirely by the attraction system, is not easy to test or prove. Research is needed into the psychology, neurobiology, endocrinology, and genetics of romantic love that merges proximate and ultimate perspectives (see Hofmann et al., 2014; Zietsch et al., 2020). It is hoped that this article, by outlining specific ways forward and means of testing the theory of co-opting mother-infant bonding will help to generate such hypotheses and research.

There are a number of things that need to occur for this new evolutionary approach to the science of romantic love to bear fruit: (i) persuade researchers that an evolutionary approach is beneficial, (ii) test the model, and (iii) extend the model. It is necessary to ensure a new generation of researchers are empowered to interpret and contribute to an evolutionary approach to romantic love. This will require new researchers to be educated about the principles, methods, and assumptions of evolutionary psychology, as well as encouragement for them to apply this knowledge to their own empirical research and theory. Many universities now have courses on evolution and human behavior, which will facilitate this. It will also be necessary to persuade established researchers in the romantic love research community that there is merit to an evolutionary approach. There are a range of resources that can assist in this respect (e.g., Confer et al., 2010; Lewis et al., 2017; Buss, 2019b). I think articles such as this one will also go some way to achieving this.

Testing the model will be multifaceted. This can be done by (i) identifying similarities and differences in psychological expression of mother-infant bonding and romantic love, (ii) identifying similarities and differences in mechanisms of mother-infant bonding and romantic love, (iii) isolating the individual components of romantic love detailed in the model, and (iv) demonstrating involvement of these components in romantic love.

Comparative psychological research between romantic love and mother-infant bonding is particularly needed. The approach taken by Leckman and Mayes (1999) may provide guidance, but alternative approaches employing variations of validated measures of attachment or preoccupation (e.g., Langeslag S. et al., 2012; Kim et al., 2013) could prove more useful (see Eastwick, 2013 for some relevant insights).

A small number of studies have attempted to identify the mechanistic similarities between mother-infant bonding and romantic love (e.g., Bartels and Zeki, 2004; Shih et al., 2022), but much more can be done. Since Bartels and Zeki’s (2004) seminal comparison between romantic and maternal love, there have been three meta-analyses of fMRI studies of romantic love (Ortigue et al., 2010; Cacioppo et al., 2012b; Shih et al., 2022), more than 30 neuroimaging studies of romantic love (Bode and Kowal, 2022), and 2 meta-analyses and at least 12 fMRI studies into maternal love (see Rigo et al., 2019; Shih et al., 2022). Additional meta-analyses of fMRI studies using different methods might find greater overlap than that found by Shih et al. (2022). A systematic review of (neuro)endocrinological studies of mother-infant bonding and romantic love would be useful. Additional genetics studies of both mother-infant bonding and romantic love are also likely to bear results.

There is a need to determine the distinct psychological and mechanistic features of each of the five components of romantic love. For example, there is a need to identify what mechanisms constitute courtship attraction. This may require neuroimaging studies of acute courtship attraction episodes or crushes, and comparing results with individuals who are in love. We need to learn what mechanisms play a role in obsessive thinking. Another meta-analysis of sexual desire and romantic love using a well-suited method would help to identify the similarities and differences between sexual desire and romantic love. Novel methods may be necessary to demonstrate involvement of the proposed components of romantic love, such as using PET to demonstrate activity of each component in all types of romantic love and at all stages (e.g., in unrequited and reciprocated love; in the initial weeks, months, and years, etc).

Further targeted neuroimaging studies could substantially contribute to a better understanding of neurobiological and neuroendocrinological systems associated with both mother-infant bonding and romantic love. A focus on maternal love of infants less than 9 months old would provide more precision to arguments related to mother-infant bonding. Region of interest fMRI studies could target regions associated with oxytocin receptors. This would be most informative comparing individuals not in a relationship with their loved one with control groups (i.e., individuals who are single and not in love or individuals who are in a relationship with their loved one). Additionally, there have been efforts to identify oxytocin receptor ligands that are useful in PET studies (e.g., Smith et al., 2016). This approach may be particularly informative, especially when investigating individuals experiencing romantic love who are not in a romantic relationship with their loved one or in the very early stages of romantic love. It would be important, in such studies, to ensure that participants are experiencing romantic love and not simply an acute courtship attraction episode. Combined PET and fMRI studies (e.g., Zürcher et al., 2021) may also provide substantial information about the relationship between neurotransmitter systems and specific neural structures in individuals experiencing romantic love.

Four regions have been specifically implicated in successful pair bonding (i.e., caudate tail, medial orbitofrontal cortex, right subcallosal cingulate, and right NA; Xu et al., 2012). Further research is needed in humans and animals to determine the way these structures promote pair bond formation. Applying an evolutionary framework to the neuroscience of romantic love will help to take into account the important human transition from promiscuous or harem-based polygynous mating to pair bonding (i.e., social monogamy or bonded polygyny) and shed light on specific mechanisms (see Cisek and Hayden, 2022) and functions (see Mundale and Bechtel, 1996 for consideration of the utility of considering a phenomenon’s function). I note that efforts have been undertaken to elucidate the manner in which pair bonding evolved from a promiscuous strategy in species with characteristics similar to humans (e.g., Gavrilets, 2012). Human neuroimaging research may also be informed by the reviews undertaken by Numan and Young (2016) and Walum and Young (2018).

I should emphasize the need for longitudinal neuroimaging, endocrinological, and genetic studies, or, at least, studies that consider time in love. If activity of the attachment system is continuous, but varies in strength or manner, it is important to map this change over time. Longitudinal studies of romantic lovers on a monthly or weekly interval until the transition from pair bond formation to pair bond maintenance or dissolution has occurred would be incredibly informative, not only to test hypotheses related to the theory of co-opting mother infant bonding, but also the trajectory of romantic love more generally. If nothing else, research into romantic love should consider the findings within a framework of pair bond formation and maintenance.

Finally, there is a need to extend the theory of co-opting mother-infant bonding. There are several details that need to be fleshed out, such as the role of mutation, natural selection, and sexual selection in the evolution of romantic love. More nuance is also needed in parts, such as the psychological differences between co-opted attraction and courtship attraction, or the roles of attachment formation and attachment maintenance in an overarching attraction system. It will be necessary to incorporate additional findings of mechanisms involved in romantic love (e.g., serotonin, nerve growth factor, cortisol; see Bode and Kushnick, 2021, for review) into the model. The specific functions of each component of romantic love and the particular psychological expressions need to be clarified. This will include ideas about the nature of the interaction between each component. There is opportunity for speculation about the sequence of co-option and recruitment. It is also necessary to ponder the role of evolution subsequent to the co-opting of mother-infant bonding and initial recruitment of courtship attraction and sexual desire. Such endeavors may drive specific hypotheses that can be tested.

## 8. Conclusion

This theoretical review presented a case for the theory of co-opting mother-infant bonding in the evolutionary history of romantic love. It attempts to generate debate about the validity of this theory and promote targeted psychological, neurobiological, endocrinological, and genetic research. First, I introduced two theories that have informed the theory of co-opting mother-infant bonding in the evolutionary history of romantic love: Fisher’s (1998) theory of independent emotions systems and the brain opioid theory of social attachment (see Machin and Dunbar, 2011). Second, I defined relevant terminology, presented a brief history of the theory of co-opting mother-infant bonding, and outlined the evidence for this theory with reference to psychological, neurobiological, and (neuro) endocrinological studies. Third, I outlined the basic premise of the theory with specific consideration of the evidence supporting the idea that romantic love involves activity of both the bonding attraction and attachment systems. Fourth, I introduced a new model of romantic love that details the evolutionary history, mechanisms, and psychological outputs of romantic love. Fifth, two unanswered questions about the evolutionary history of romantic love were posed. I concluded with ideas for future research. The result is the articulation of a theory that may partially account for the evolutionary history of romantic love and serve as a basis for a new approach to the science of romantic love. “Nothing in biology makes sense except in the light of evolution” (Dobzhansky, 1973, p. 125). I would suggest that everything in the science of romantic love makes more sense in the light of evolution.

## Author contributions

The author confirms being the sole contributor of this work and has approved it for publication.
