# Corrigendum: Romantic love evolved by co-opting mother-infant bonding

**DOI:** 10.3389/fpsyg.2024.1402313

**Published:** 2024-04-29

**Authors:** Adam Bode

**Affiliations:** School of Archaeology and Anthropology, ANU College of Arts and Social Sciences, The Australian National University, Canberra, ACT, Australia

**Keywords:** attachment, attraction, co-option, evolution, mother-infant bonding, pair bonding, romantic love

In the published article, there were errors in the text. Firstly, it was falsely stated that the VTA contains oxytocin receptors.

A correction has been made to *3.2. A brief history of the theory of co-opting mother-infant bonding, Paragraph 2*.

This sentence previously stated:

“That meta-analysis highlighted similarities between maternal and romantic love in the dopamine and oxytocin-rich left VTA.”

The corrected sentence appears below:

“That meta-analysis highlighted similarities between maternal and romantic love in the dopamine-rich left VTA.”

Secondly, there were five instances (in four sentences) when an incorrect citation was provided. The incorrect citation was Marazziti et al. (2014). The correct citation is Marazziti et al. (2021).

The following corrections have been made to section *4.1. The basic premise*.

This first incorrect sentence, found in *Paragraph 1*, previously stated:

“These features of infatuation may be more common when the dopaminergic activity of mate choice mechanisms (i.e., attraction) are active without substantial calmative effect of the oxytocinergic attachment system (see Marazziti et al., 2014).”

The corrected sentence appears below:

“These features of infatuation may be more common when the dopaminergic activity of mate choice mechanisms (i.e., attraction) are active without substantial calmative effect of the oxytocinergic attachment system (see Marazziti et al., 2021).”

This second incorrect sentence, found in *Paragraph 2*, previously stated:

“Whereas Fisher et al. (2016) and others (e.g., Marazziti et al., 2014) believe that romantic love precedes a period of pair bonding, I assert that part of romantic love is the process of pair bonding (i.e., pair bond formation).”

The corrected sentence appears below:

“Whereas Fisher et al. (2016) and others (e.g., Marazziti et al., 2021) believe that romantic love precedes a period of pair bonding, I assert that part of romantic love is the process of pair bonding (i.e., pair bond formation).”

This third incorrect sentence, found in *Paragraph 3*, previously stated:

“Marazziti et al. (2014) describe a process in which oxytocin is produced in the hypothalamus (one of the regions where attraction is generated) and that transforms anxiety/fear reactions into a sense of “well-being, reward, and joy” (p. 252).”

The corrected sentence appears below:

“Marazziti et al. (2021) describe a process in which oxytocin is produced in the hypothalamus (one of the regions where attraction is generated) and that transforms anxiety/fear reactions into a sense of “well-being, reward, and joy” (p. 252).”

This fourth incorrect sentence, found in *Paragraph 4*, previously stated:

“Unlike the model proposed by Marazziti et al. (2014), Fisher (1998, 2000), and Fisher et al. (2002), the theory of co-opting mother-infant bonding suggests that oxytocinergic activity is a necessary component of romantic love, and the “second step” that Marazziti et al. (2014) refer to is, in fact, part of romantic love.”

The corrected sentence appears below:

“Unlike the model proposed by Fisher (1998, 2000), Fisher et al. (2002), and Marazziti et al. (2021), the theory of co-opting mother-infant bonding suggests that oxytocinergic activity is a necessary component of romantic love, and the “second step” that Marazziti et al. (2021) refer to is, in fact, part of romantic love.”

The author apologizes for these errors and states that they do not change the scientific conclusions of the article in any way. The original article has been updated.
